# Fibroblast, Epithelial and Endothelial Cell-Derived Human Cytomegalovirus Strains Display Distinct Neutralizing Antibody Responses and Varying Levels of gH/gL Complexes

**DOI:** 10.3390/ijms24054417

**Published:** 2023-02-23

**Authors:** Chiara Fornara, Eric Schultz, Daniele Lilleri, Fausto Baldanti, Brent Ryckman, Giuseppe Gerna

**Affiliations:** 1 Microbiology and Virology Unit, Fondazione IRCCS Policlinico San Matteo, 27100 Pavia, Italy; 2 Center for Biomolecular Structure and Dynamics, University of Montana, Missoula, MT 59812, USA; 3 Division of Biological Sciences, University of Montana, Missoula, MT 59812, USA; 4 Department of Clinical, Surgical, Diagnostic and Pediatric Sciences, University of Pavia, 27100 Pavia, Italy; 5 Laboratories of Genetics, Transplantology and Cardiovascular Diseases, Fondazione IRCCS Policlinico San Matteo, 27100 Pavia, Italy

**Keywords:** human cytomegalovirus, primary infection, neutralizing antibodies, trimer, pentamer, receptors

## Abstract

In sequential sera from pregnant women with HCMV primary infection (PI), the serum neutralizing activity is higher against virions produced in epithelial and endothelial cells than in fibroblasts. Immunoblotting shows that the pentamer complex/trimer complex (PC/TC) ratio varies according to the producer cell culture type used for the virus preparation to be employed in the neutralizing antibody (NAb) assay, and is lower in fibroblasts and higher in epithelial, and especially endothelial cells. The blocking activity of TC- and PC-specific inhibitors varies according to the PC/TC ratio of virus preparations. The rapid reversion of the virus phenotype following its back passage to the original cell culture (fibroblasts) potentially argues in favor of a producer cell effect on virus phenotype. However, the role of genetic factors cannot be overlooked. In addition to the producer cell type, the PC/TC ratio may differ in single HCMV strains. In conclusion, the NAb activity not only varies with different HCMV strains, but is a dynamic parameter changing according to virus strain, type of target and producer cells, and number of cell culture passages. These findings may have some important implications for the development of both therapeutic antibodies and subunit vaccines.

## 1. Introduction

In the last decades, the neutralizing antibody (NAb) response during human cytomegalovirus (HCMV) primary infection (PI) has been investigated by several groups. In the 1990s, gB and gH were considered the glycoprotein complexes (gCs) responsible for eliciting most of the HCMV neutralizing activity [1,2,3]. Subsequently, the gM/gN glycoprotein complex was shown to be an important target of the strain-specific NAb response [4,5]. Finally, another envelope glycoprotein complex, gCIII (gHgL), was shown to be associated with either gO, thus forming the trimer complex (TC) gHgLgO [6], or pUL128L (*UL131-128* locus), thus giving rise to the pentamer complex (PC) gHgLpUL128L [7,8]. TC binds to platelet-derived growth factor receptor-α (PDGFR-α), mediating the HCMV entry into diploid human embryonic lung fibroblasts (HELF) [9], while PC binds to neuropilin-2 (Nrp-2) and mediates entry into epithelial/endothelial cells [10].

When convalescent sera from PI were absorbed with purified gCs, sera absorbed with PC showed a >90% reduction in the NAb capacity on epithelial cells, whereas the NAb capacity was unmodified in sera preabsorbed with gHgL or gB [11]. Furthermore, antibodies to PC were shown to be mostly neutralizing (Nt), unlike antibodies to gB, which were shown to be mostly non-Nt [12]. In addition, HCMV hyperimmune Ig preparations were shown to comprise the majority of NAb, which were directed to PC [13]. Thus, in the last decade, NAb directed to PC, and in particular to pUL128L, have been considered responsible for most of the Nt activity of HCMV-specific humoral immune response [12,13,14]. Recently, both TC- and PC-specific antibodies have been shown to share a comparable level of neutralizing potency and to act in a synergistic way to neutralize HCMV both in transplant recipients and pregnant women as well as to prevent HCMV cell-to-cell spread [15].

Furthermore, when tested in a group of pregnant women with PI, NAb preventing HCMV infection of epithelial/endothelial cells were detected at higher titers and earlier than NAb preventing HCMV infection of HELF cells [16,17]. HCMV strains vary in the assembly of TC and PC into virion envelopes and this can influence virion infectivity on both fibroblasts and epithelial cells [18,19]. By examining some well-known HCMV strains, such as Merlin, TR, and TB40/E, it was found that Merlin virions contain more PC than TC, whereas TR and TB40/E contain much more TC than PC. In general, the infectivity of each strain correlated with the amount of TC. These and other studies documented that, while the TC was required for virus entry into all cell types [6,20], PC was critical for infection only of epithelial/endothelial cells as well as monocytes/macrophages and dendritic cells [7,21]. More recently, both TC and PC have been reported to promote a two-step process for HCMV entry into epithelial/endothelial cells [22]. The differential neutralizing potency of anti-HCMV sera against virus preparations produced in different cell types appears worthy of investigation.

Following the identification of the PDGFR-α as the TC receptor for infection of HELF [9], the PC receptor for infection of epithelial/endothelial cells was identified as the Nrp-2 [10]. However, while Nrp-2 was found to play a predominant role for interacting with PC in the infection of epithelial/endothelial cells, both PDGFR-α and Nrp-2 receptors were found to mediate infection of HELF, with PDGFR-α playing a major role [10]. Briefly, TC was found to interact with PDGFR-α to guide the entry into HELF, while involving macropinocytosis with pH-independent cell membrane fusion. On the other hand, PC would interact with Nrp-2 in an entry process into epithelial/endothelial cells involving endocytosis and a low pH-dependent fusion [23]. In addition to PDGFRα, TGFβRIII has been shown to bind in a mutually exclusive manner to TC, although its role in entry is still unknown [24].

The aforementioned findings that anti-HCMV sera display cell-type dependent neutralization potency [17] and the recent identification of the major HCMV TC- and PC-specific receptors interacting with their respective ligands in HELF and epithelial/endothelial cells, prompted us to conduct a series of cross-neutralization (cross-Nt) experiments among sequential sera collected from three pregnant women with HCMV PI in order to investigate the relationship of the NAb potency with the TC and PC expression as well as the producer cell effects on the three HCMV isolates grown in different cellular systems. As for the producer cell effects, i.e., that difference in physiology among different cell types can affect the assembly properties of the virions released, this concept was first introduced for EBV glycoproteins gH/gL and gH/gL/gp42 [25]. Although they are not well understood or described, there are probably producer cell effects also for HCMV [26,27].

As a conclusion, we found that, in addition to the time point of serum collection after PI onset, the NAb activity showed a great level of variation according to the following factors: (i) type of cell culture (HELF, epithelial, or endothelial cells) used for cell-free virus preparations to be employed in the NAb assays; (ii) the cell type used for NAb assay assessment; (iii) HCMV strain responsible for each case of PI (i.e., NAb titer was higher when measured against the strain having actually caused the infection in the patient), its cell culture passage history and the PC/TC expression ratio.

## 2. Results

In order to facilitate the analysis of the correlation between NAb titers and distribution of TC and PC in different virus preparations, results will be reported as follows: first, NAb titers; second, PC-specific and TC-specific inhibition; and, third, immunoblotting assays to illustrate the gC distribution.

### 2.1. NAb Titer of Sequential Sera from 3 Patients (p-232, p-236, and p-237) vs. Homologous and Heterologous HCMV Strains (VR#1, VR#2, and VR#3, Respectively)

Following isolation [28,29,30] and propagation of VR#1, VR#2, and VR#3 in HELF, cell-free virus preparations for use in cross-Nt assays were obtained for each of the three virus strains from HELF cells. As a result, the following three virus preparations were obtained (the number after the cell culture acronym indicates the number of passages on the same cell culture): VR#1 HELF/14; VR#2 HELF/12; and VR#3 HELF/12 (Table 1, Table 2 and Table 3). Subsequently, each virus was propagated for a variable number of passages in epithelial (ARPE-19) or endothelial cell (HUVEC) cultures until preparation of cell-free virus stocks from either type of producer cells with sufficient infectious titer to be used in cross-Nt assays. For ARPE-19, virus preparations were as follows: VR#1 HELF/8 ARPE/3; VR#2 HELF/9 ARPE/4; and VR#3 HELF/12 ARPE/4 (Table 1, Table 2 and Table 3). On the other hand, HUVEC preparations were as follows: VR#1 HELF/11 HUVEC/20; VR#2 HELF/4 HUVEC/14; and VR#3 HELF/10 HUVEC/8 (Table 1, Table 2 and Table 3).

Initially, the gB, gH and gO genotypes of the three virus isolates were determined. VR#1 was classified as gB1 gH1 gO4; VR#2 as gB1 gH2 gO2b; VR#3 as gB2 gH1 gO1b; the reference strain VR1814, used in some experiments, was classified as gB3 gH1 gO1c [31,32]. 

Both homologous and heterologous NAb titers, as determined in HELF target cells, were negative or weakly positive (<1:10–1:20) within the first 30–60 d after the onset of PI (Table 1, Table 2 and Table 3). However, a year thereafter, both homologous and heterologous NAb titers were positive in HELF, with the homologous titer 8–32-fold higher than the heterologous titers. As an example, p-232 was negative for both homologous (VR#1) and heterologous (vs. VRs #2 and #3) NAb titer at 60 d, then becoming slightly positive (1:10) at 90 d and markedly positive at 360 d, with homologous (vs. VR#1) NAb titer 16–32-fold higher than heterologous titers (vs. VR#2, and VR#3). NAb titer could not be determined in either ARPE-19 or HUVEC target cells, when directly using virus preparations from HELF producer cells upon first passage, since HELF-produced virus preparations were poorly infectious for ARPE-19 or HUVEC target cells (Table 1, Table 2 and Table 3).

On the contrary, following a few passages in ARPE-19 epithelial producer cells, virus preparations were readily available for both homologous and heterologous NAb titer determination. Both titers were already positive in HELF target cells (≥1:10), ARPE-19 epithelial target cells (≥1:160) and HUVEC target cells (≥1:20) within 60 d after PI onset. A year thereafter, homologous and heterologous NAb titers were both markedly positive (≥1:640). In addition, using virus preparations from ARPE-19 producer cells, both homologous and heterologous NAb titers were mostly able to detect seroconversion (referred to as passage of NAb titer from seronegative to seropositive or from seropositive to ≥ fourfold rise) in nearly all three test systems, except for the homologous NAb titer measured on ARPE-19 target cells. In other words, the only cases where seroconversion was sometimes not detected, were in ARPE-19 cells showing a very high/stable NAb titer throughout follow-up since the beginning (see VR#1 and VR#3 on ARPE-19 cells, Table 1 and Table 3).

Similarly, both homologous and heterologous, NAb titers vs. virus strains recovered in HELF and propagated in HUVEC producer cells were consistently positive (≥1:40) in HUVEC target cells within 60 days after PI onset. A year thereafter, the homologous NAb titer was 4–32-fold higher than the heterologous titer. As for seroconversion, it could be detected with both homologous and heterologous NAb titer in HELF and ARPE-19, while sometimes it could not be observed in HUVEC (Table 1, Table 2 and Table 3).

### 2.2. NAb Titers vs. VR#1814

In addition, we used, as our reference HCMV strain, VR#1814, which was originally recovered from cervical secretions of a healthy pregnant woman [33]. VR#1814, following initial propagation in HELF (HELF/27), and subsequent extensive propagation in HUVECs (HUVEC/132, HUVEC/455), consistently maintained its competence for growth in HUVECs and its transfer capacity to leukocytes (thus, referred to as Huv^+^Leuk^+^). Similarly, its propagation in ARPE-19 cells consistently preserved its Huv^+^Leuk^+^ properties. Furthermore, its cloning as a bacterial artificial chromosome (BAC) documented that the locus *UL128L* was indispensable for the maintenance of the above reported properties [7]. Following 132 previous passages in HUVEC, our reference strain VR#1814 was propagated eight times in ARPE-19 producer cells. In addition, we recovered the original virus isolate, which had been previously propagated 27 times in HELF producer cells prior to testing it in the three cell culture targets. The number of passages varied according to the need for obtaining the titer of cell-free virus preparation required for performing the NAb assay. As shown in Table 4, NAb titers vs. VR#1814 of the three sequential serum sample series from three subjects with PI were in HELF comparable to those observed when VR#1, VR#2, and VR#3 were propagated in HELF producer cells (sometimes with some delay in the detection of seroconversion). In contrast, when VR#1814 cell-free virus preparations were obtained in ARPE-19 or HUVEC producer cells, NAb were already detected for either epithelial or endothelial target cells at the beginning of follow-up with lack of detection of seroconversion. In conclusion (Table 4), these findings seem to indicate that the NAb response may be detected much earlier after PI onset when using virus propagated in epithelial or endothelial producer cells (early seroconversion) than virus propagated in fibroblast producer cells (late seroconversion).

### 2.3. Residual HCMV Infectivity (VR#1 to VR#3) following NAb Inhibition by PC-Specific and TC-Specific Inhibitors in Different Cell Cultures

Using serial concentrations of PC-specific and TC-specific inhibitors against the three virus preparations obtained for each virus (VR#1, VR#2 and VR#3, respectively) from the three producer cell culture systems employed (HELF, ARPE-19 and HUVEC), it was possible to quantify the relevant activity (IC_50_) of the different inhibitory agents. This activity was variable in the different preparations of each virus according to the producer cell system adopted.

**Figure 1:**
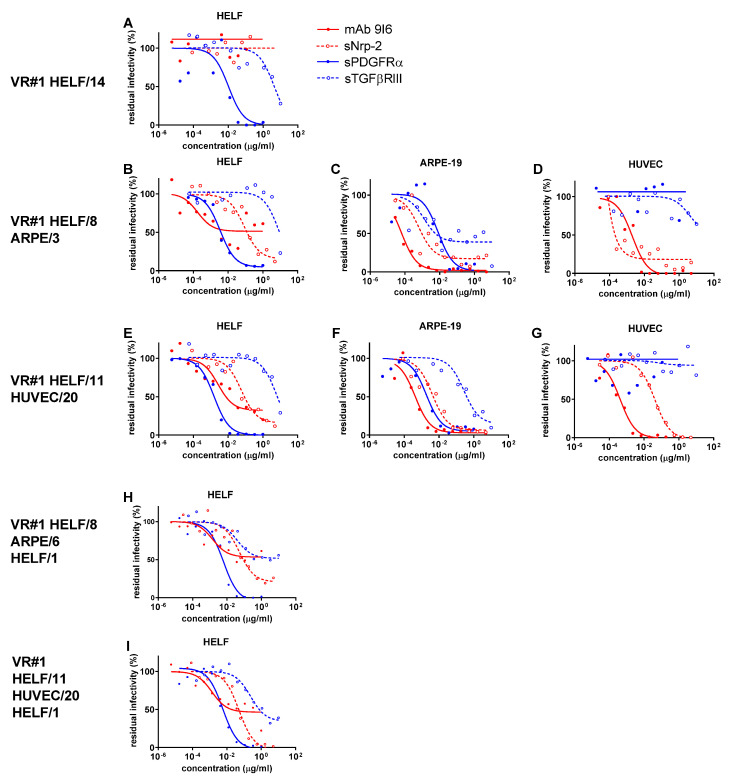
ICs_50_ of anti-PC (mAb 9I6, and sNrp-2) and anti-TC (sPDGFR-α, and sTGFβRIII) activity in inhibiting infectivity of VR#1, which was recovered and propagated in HELF/14 (**A**), then passaged in ARPE-19/3 (**B**–**D**) cells, and finally, in HUVEC/20 (**E**–**G**) primary cells, as determined in HELF (**A**,**B**,**E**), ARPE-19 (**C**,**F**) and HUVEC (**D**,**G**) cell substrates against residual infectivity. The inhibiting activities of VR#1 infectivity by different agents following back passages of VR#1 from ARPE/16 and HUVEC/20 onto HELF/1 are shown in (**H**,**I**), respectively.

**Figure 2:**
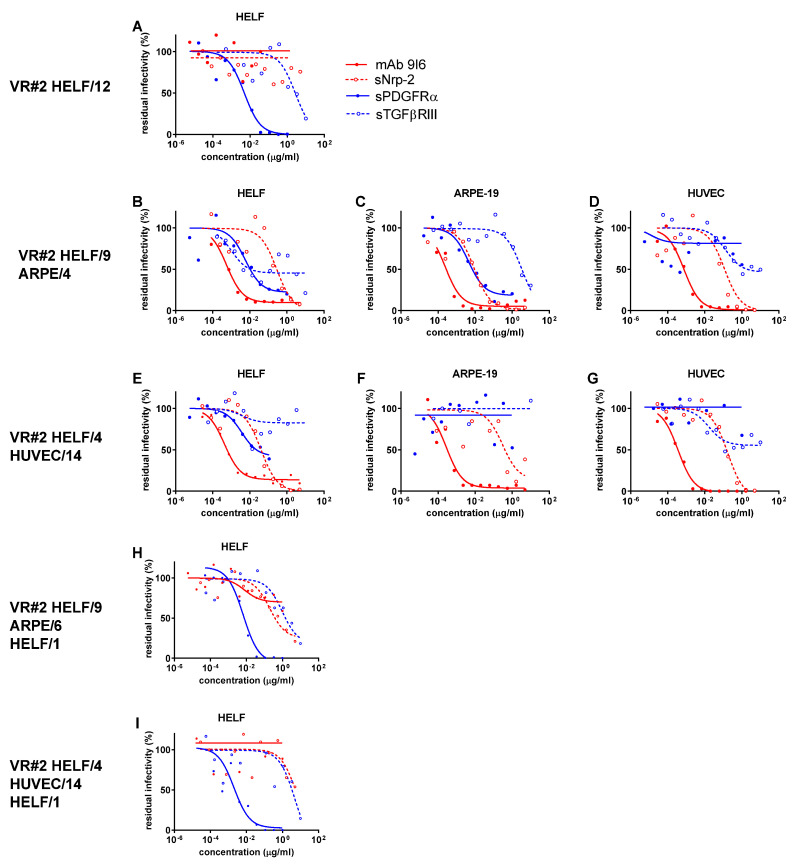
ICs_50_ of anti-PC (mAb 9I6, and sNrp-2) and anti-TC (sPDGFR-α, and sTGFβRIII) activity in inhibiting infectivity of VR#2, which was recovered and propagated in HELF/12 (**A**), then passaged in ARPE-19/4 (**B**–**D**) cells, and finally, in HUVEC/14 (**E**–**G**) primary cells, as determined in HELF (**A**,**B**,**E**), ARPE-19 (**C**,**F**) and HUVEC (**D**,**G**) cell substrates against residual infectivity. In addition, back passages from ARPE/6 to HELF/1 (**H**) and from HUVEC/14 to HELF/1 (**I**) are reported.

**Figure 3:**
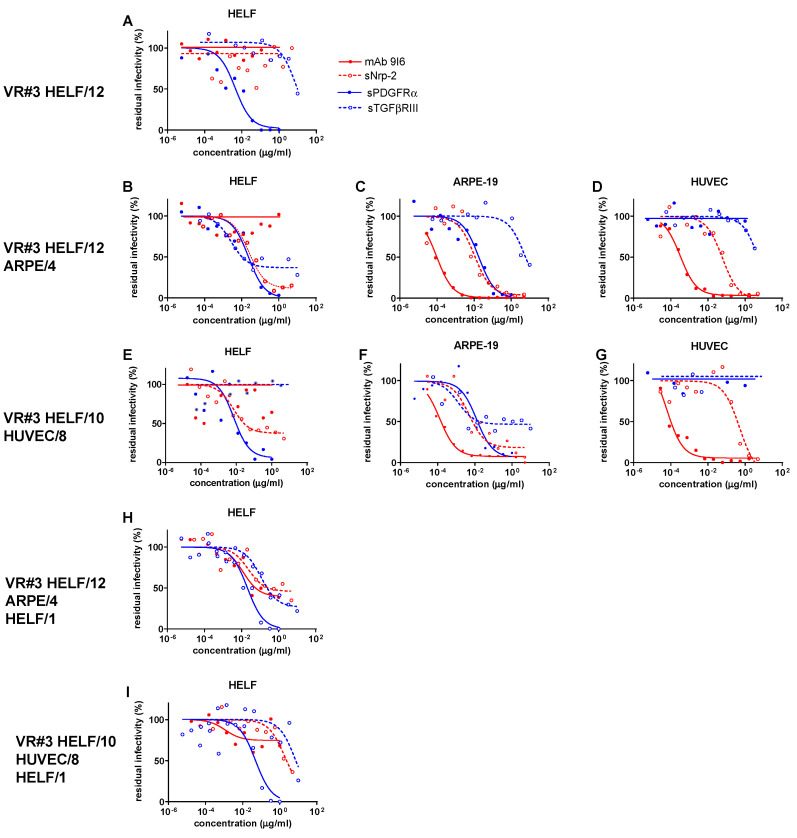
ICs_50_ of anti-PC (mAb 9I6, and sNrp-2) and anti-TC (sPDGFR-α, and sTGFβRIII) activity in inhibiting infectivity of VR#3, which was recovered and propagated in HELF/12 (**A**), then passaged in ARPE-19/4 (**B**–**D**) cells, and finally, in HUVEC/8 (**E**–**G**) primary cells, as determined in HELF (**A**,**B**,**E**), ARPE-19 (**C**,**F**) and HUVEC (**D**,**G**) cell substrates against residual infectivity. In addition, the inhibiting activities by different agents following back passages of VR#3 from ARPE/4 and HUVEC/8 onto HELF/1 are shown in (**H**,**I**), respectively.

As shown in Table S1 and Figure 1A, Figure 2A and Figure 3A, all three virus preparations (VR#1 to VR#3) produced in HELF (passages 12–14) were entirely blocked (within the inhibitor concentrations used) in their potential infectivity in HELF by PDGFRα (but not by TGFβRIII), whereas no IC_50_ could be determined for the PC-specific mAb and Nrp-2 soluble receptor (no blocking activity determined). However, when examining the blocking activity of the four inhibitors on the virus preparations of VR#1 to VR#3 produced after a few passages in ARPE-19 epithelial cells (Figure 1B–D, Figure 2B–D and Figure 3B–D), the ICs_50_ of PDGFRα as well as of the α-PC mAb and Nrp-2 were well determined for VR#2 as well as for VR#1 and VR#3. In particular, VR#2 infection of HELF was almost completely neutralized by anti-PC mAb, (with an IC_50_ similar to that observed in ARPE-19 and HUVEC, Figure 2B–D), and, to a lesser extent, by Nrp-2. On the other hand, the effect of anti-PC mAb and Nrp-2 on VR#1 and VR#3 preparations, was less pronounced. As for TGFβRIII, the inhibitory activity was minimal, if not absent. When the virus preparations produced in ARPE-19 were tested in HUVEC, no activity was detected for PDGFRα or TGFβRIII in any of the three virus preparations (no blocking), whereas IC_50_ for both α-PC mAb and Nrp-2 were well determined (Figure 1D, Figure 2D and Figure 3D). Finally, the analysis of the blocking activity of the four inhibitors on the virus preparations produced and tested in HUVEC, indicated that the blocking effect was due exclusively to mAb to PC, and, to a lesser extent, to Nrp-2 (Figure 1G, Figure 2G and Figure 3G).

To preliminarily investigate whether changes in neutralization occur rapidly and are the result of producer cell effects, or reflect genetic adaptation to epithelial/endothelial cells, cell-free virus preparations from ARPE-19 and HUVEC cells underwent a single passage back to HELF at a multiplicity of infection (MOI) of 0.1–1.0. As a result, the viral characteristics reverted in all cases. What is interesting is that passage back to HELF (HELF/1) of VR#2 from ARPE/6 or HUVEC/14 reconstituted substantially the serum Nt activity (Table S2) as well as the only major inhibiting activity of PDGFRα similar to that observed with the original HELF-produced virus preparation (HELF/12) following virus recovery from the clinical sample (Figure 2H,I). A similar, but more attenuated Nt trend, was observed for VR#1 and VR#3 from ARPE or HUVEC back to HELF. This reversion of neutralization characteristics after a single back passage to HELF argues against genetic mutations affecting the expression of TC/PC during epithelial/endothelial cell passages. In addition, the lower reversion process mentioned for VR#1 and VR#3 might also be due to the different genotypes reported above among the three virus strains.

**Figure 4:**
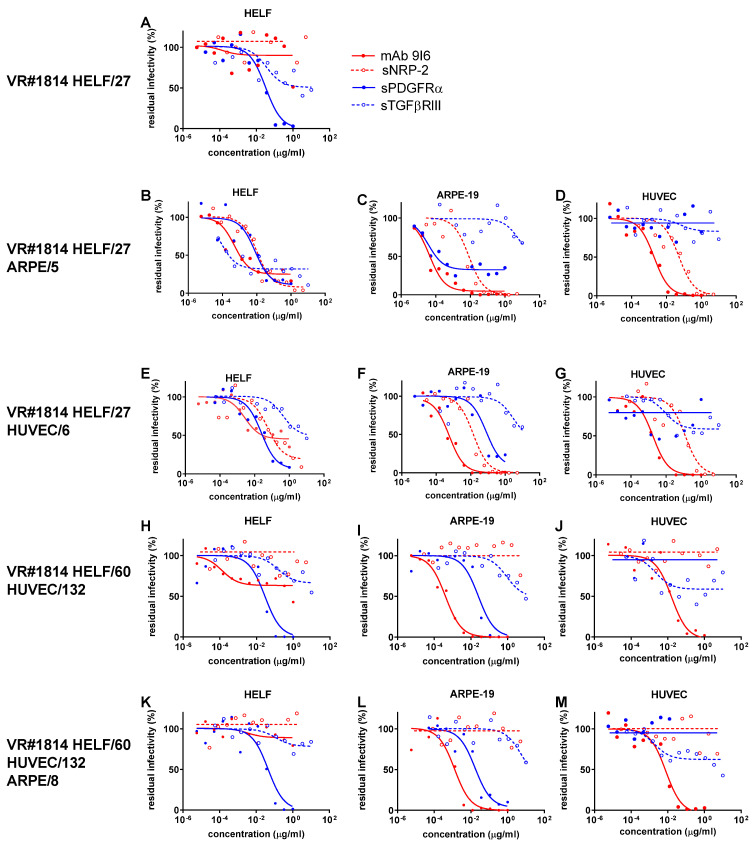
ICs_50_ of anti-PC (mAb 9I6, and sNrp-2) and anti-TC (sPDGFR-α and sTGFβRIII) activity in inhibiting infectivity of reference strain VR#1814, which was recovered in HELF from cervical secretions and propagated in HELF/27 (**A**), and then in HELF/27 ARPE/5 (**B**–**D**) and HELF/27 HUVEC/6 (**E**–**G**), and finally passaged in HELF/60 HUVEC/132 (**H**–**J**) primary cells, and then, in HELF/60 HUVEC/132 ARPE/8 (**K**–**M**). ICs_50_ of different inhibitors were determined for each passage in HELF (**A**,**B**,**E**,**H**,**K**), ARPE-19 (**C**,**F**,**I**,**L**), and HUVEC (**D**,**G**,**J**,**M**) cell substrates against residual infectivity.

As for our reference strain VR#1814, after a high number of passages in fibroblasts (HELF/60) and endothelial cells (HUVEC/132), a major blocking effect on the three types of virus preparations produced by the three types of cell cultures was shown by: (i) PDGFRα, when inhibitory activity was tested in HELF (Figure 4H); (ii) both PDGFRα and mAb anti-PC, when inhibition was tested in ARPE-19 (Figure 4I); (iii) mAb anti-PC, when inhibitory agents were tested in HUVEC (Figure 4J). The other inhibitors displayed much lesser (or absent) activity. In particular, the lack of blocking activity by Nrp-2 was observed for VR#1814 HUVEC/132 passage (Figure 4H–J) as well as its subsequent ARPE/8 passage (Figure 4K–M). Similarly, a total lack of inhibiting activity by Nrp-2 was found for VR#1814 HUVEC/455 virus preparation. However, in all three cell systems, when low-passaged VR#1814 from HELF/27 was propagated in ARPE-19 (ARPE/5, Figure 4B–D) or HUVEC (HUVEC/6, Figure 4E–G), block of VR#1814 by Nrp-2 was complete.

### 2.4. Expression of PC and TC in Different Virus Preparations

**Figure 5:**
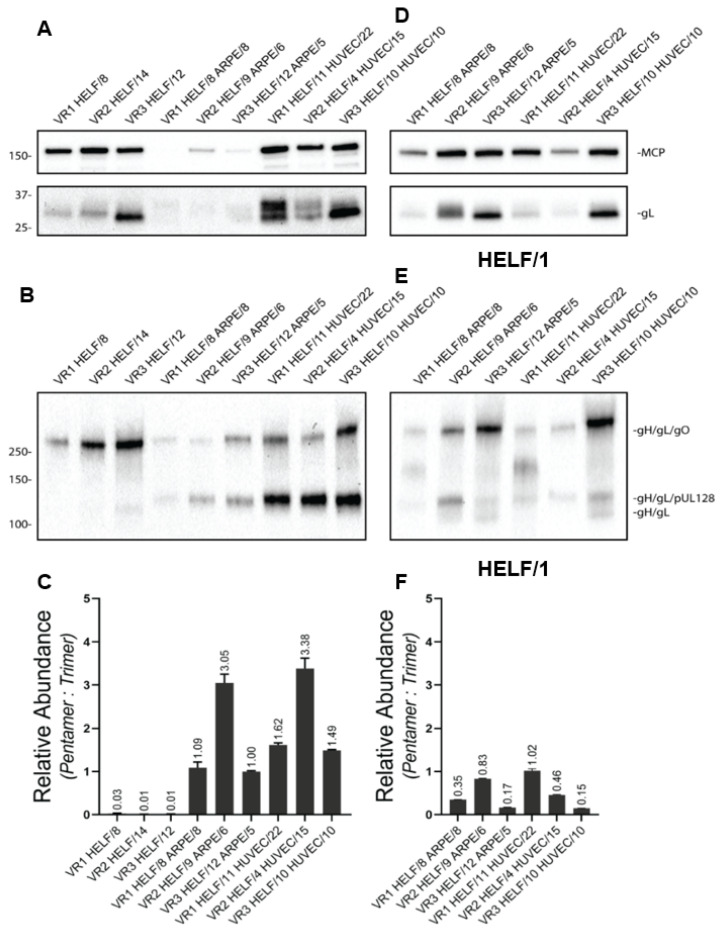
Immunoblot analysis of: (**A**–**C**) cell-free VR#1 to VR#3 virus preparations on human fibroblasts (HELF), epithelial (ARPE-19) and endothelial cells (HUVEC); and (**D**–**F**) the same three virus preparations after a single back passage from ARPE-19 and HUVEC to HELF. (**A**,**D**): reducing conditions. (**B**,**E**): non-reducing conditions. (**C**,**F**): PC/TC ratio.

To determine whether TC or PC was the predominant gH/gL complex in the virus preparations, immunoblot analysis was performed. Under reducing conditions (Figure 5A), VR#3-HELF showed a higher amount of total gL (i.e., PC or TC) than VR#1-HELF and VR#2-HELF; among HUVEC preparations, VR#3 and VR#1 showed higher amount of total gL than VR#2. Non-reducing gels (Figure 5B) showed that in the HELF-derived virus preparations all the gL was in the form of the TC. In HUVEC and ARPE-19 virus preparations, gL was present both in the form of TC and PC: VR#1 andVR#3 showed a similar amount of the two complexes in ARPE-19 and a slightly higher amount of PC than TC in HUVEC, while in VR#2 the PC was much more abundant than TC in both ARPE-19 and HUVEC (Figure 5C). After a single back passage of the three virus strains from ARPE-19 and HUVEC to HELF, under non-reducing conditions the PC/TC ratio was markedly reduced (Figure 5E,F vs. Figure 5B,C, respectively), though not to the level of HELF-isolated viruses (Figure 5B,C).

### 2.5. ELISA IgG Antibody Response to gCs

**Figure 6:**
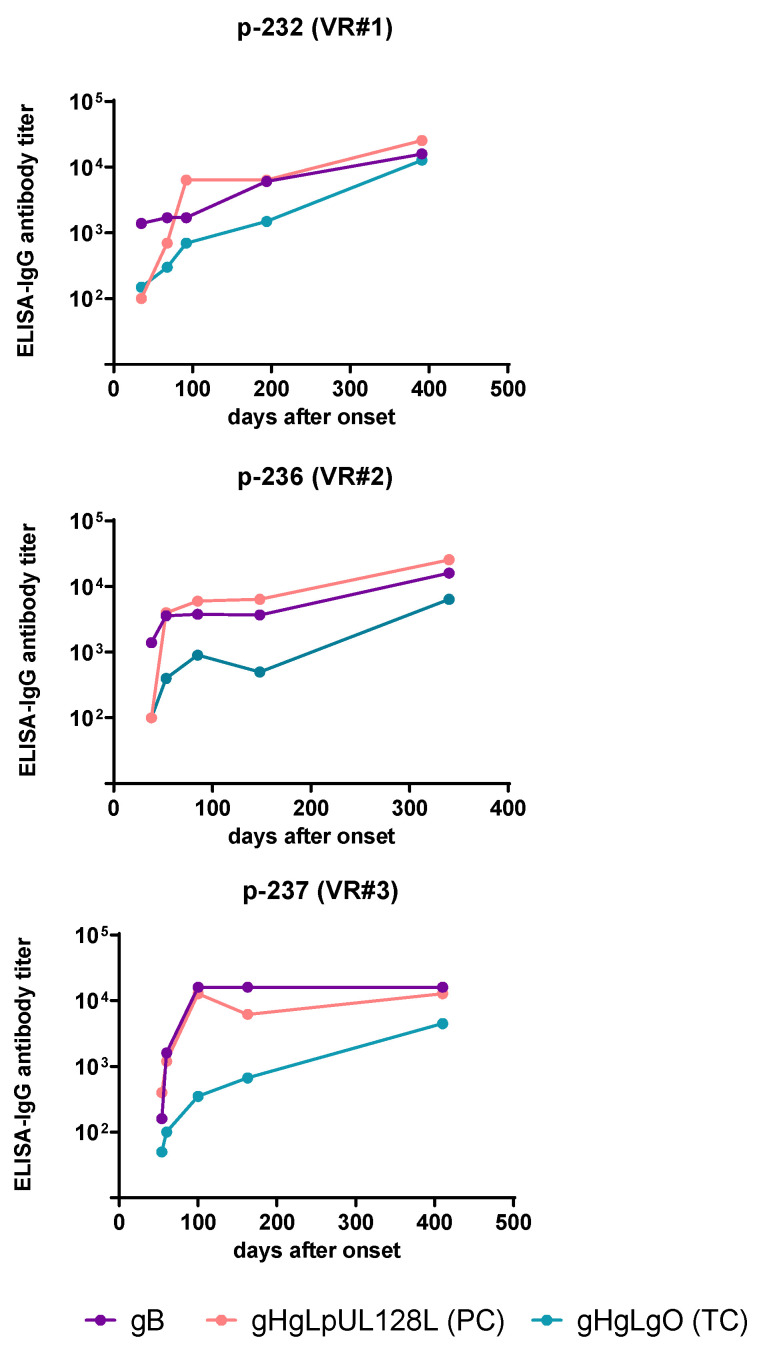
ELISA IgG antibody titer to gB, PC (gHgLpUL128L), and TC (gHgLgO) in five sequential serum samples collected from three pregnant women (p-232, p-236 and p-237) with PI during the first year after PI onset. For each curve, the five colored dots indicate the five time points of blood collection.

Based on the previously documented correlation between IgG-pentamer antibody titers measured by ELISA and NAb titers measured in ARPE-19 epithelial cells within 60 d after PI onset, and the previous conclusion that most of the Nt activity detected in sera from pregnant women with PI is conferred by PC NAb [11], we decided to test the ELISA IgG Ab titers against the three HCMV gCs (PC, TC, and gB) in the three series of five sequential serum samples from the three pregnant women with PI. As shown in Figure 6, using an ELISA IgG antibody assay previously developed for the determination of the antibody response to HCMV gB, PC, and TC gCs [11], we observed that within 60 d after PI onset, gB antibodies were already present in blood of the three pregnant women with PI, while antibodies to PC and TC started appearing (ser#1). At 60–90 d antibodies to gB and PC were consistently present, whereas antibodies to TC were, when present, very low in titer (ser#2). At 90–120 d antibodies to gB and PC reached high titers (ser#3), which persisted until 6 months p.i., while antibodies to TC persisted at median-low titers in the 3–6 month time lapse p.i. (ser#4). Finally, at 360 d p.i. antibodies to gB and PC reached the highest titers, as well as antibodies to TC, which however were consistently lower in titer (ser#5).

## 3. Discussion

The key points of the conclusions of our study are as follows: (i) the correlation of the NAb titer detected and the PC/TC ratio of the viral preparation adopted in the assay; (ii) the variable distribution of gCs TC and PC according to the cell system used for virus propagation, as shown by both inhibition and immunoblotting assays; (iii) the importance of defining the antigenic properties of each virus preparation in view of adopting either clinical or preventive measures.

Our study documents that the NAb response mostly correlates with inhibition of: (i) TC in HELF; (ii) both TC and PC in ARPE-19; (iii) PC in HUVEC. Immunoblotting analysis showed that, although PC was not detected in HELF virus preparations of VR#1 to VR#3, following some passages in ARPE-19 cells and HUVEC, PC was detected in high amounts in virus preparations of VR#1 and VR#3, and was predominant compared to TC in VR#2 preparations. The relative contribution of epigenetic factors (so called “producer cell effects”) and rapid genetic adaptation [34,35,36] in the determination of PC and TC content of the HCMV virions remains debated, although the quick recovery of PC in ARPE-19 and HUVEC suggests that the producer cell effects may play a significant role. It must be taken into consideration that these changes could be the result of genetic adaptation to culture, selection/propagation of preexisting genotypic variants, or epigenetic factors between cell types. Moreover, the potential role of different genotypes cannot be ruled out. However, in the absence of sequencing, the genetic role cannot be determined in our study.

The analysis of the blocking activity of the selective inhibitors of PC (mAb 9I6 [11,14,37] and Nrp-2 receptor) and TC (soluble PDGFR-α and TGFβRIII) shows that HCMV uses three different mechanisms to enter HELF, ARPE-19 or HUVEC cells. As for mAb 9I6, it was reported to be specific for p-UL128L, while a second mAb specific for p-UL130-131 [14] behaved in a comparable way. Thus, two PC-specific mAbs reactive with different epitopes gave similar blocking results. Entry into HELF is mostly dependent on TC, but also PC might be involved, although to a lesser extent, as already reported [10]. In addition, ARPE- and HUVEC-derived HCMV strains show a variable usage of PC to enter HELF: VR#2 used both TC and PC at a comparable level; VR#1 used mainly TC with a limited usage of PC, while usage of PC was almost negligible for VR#3. This may be explained by the different PC/TC ratio expression in HUVEC- and ARPE-derived virus preparations, as shown by immunoblotting: VR#2 expressed PC at higher level than TC, whereas VR#1 and VR#3 expressed PC and TC at similar levels. Entry into ARPE-19 cells is dependent on both PC and TC, since both inhibitors block completely the infection of these cells. Finally, entry into HUVEC is dependent on PC only, whereas TC is not or very partially involved, as shown by nearly lack of blocking activity of TC inhibitor(s). However, these conclusions must be questioned by the potential role of interfering non-specific factors, such as the fact that mAbs and soluble ligands can block virus infection by non-specific mechanisms such as steric hindrance. In addition, more players can be involved in virus infectivity than TC and PC [27]. It is tempting to speculate that different TC/PC ratios of different virus isolates, as detected in first HELF passages after virus isolation, may be somewhat related to the site of virus recovery and cell types infected in vivo.

Some results of our study were already reported more than a decade ago, when it was shown that in HCMV PI NAb detected in HELF took about three months to appear, whereas NAb measured in endothelial/epithelial cells were detected within 10 d after PI onset [17]. These results have now been confirmed and may now be better interpreted in the light of the recent discovery of HCMV receptors of HELF and epithelial/endothelial cells. In HCMV PIs, NAb have been found to be detectable late (after 3–6 months) in HELF cells [16]. Since antibodies to TC appear later than antibodies to PC and infection of HELF is mainly dependent on TC interaction with PDGFRα [9], NAb are detected only late in HELF. On the other hand, NAb to PC appear much earlier and may be detected 10 d after PI onset in ARPE-19 cells and HUVEC. This is because they appear very early and prevent binding of PC to its major receptor, which has been shown first to be the Nrp-2 [10] and then the olfactory receptor OR14I1 [38] in epithelial/endothelial cells. Thus, a human serum in the convalescent phase of a HCMV PI is a mixture of NAb to TC (detectable late in HELF cells), and PC (detectable very early in epithelial/endothelial cells).

In addition to these two primary receptors, other co-receptors (or accessory receptors or host proteins) are likely to be involved in virus entry. Recently, as mentioned above, Kschonsak et al. [24], by showing by cryo-electron microscopy that TC can bind in a mutually exclusive way to either PDGFRα or TGFRβRIII, documented that both receptors may act as independent receptors. In the present study, the inhibitory activity of TC by TGFβRIII was found to be much less potent than that of PDGFRα after virus propagation in HELF and ARPE-19 cells, and nearly absent (as that of PDGFRα) after virus propagation in HUVEC. Differences in the blocking activity of inhibitory agents may depend on the concentration used. In our study, the substantial lack of inhibitory effect of Nrp-2 in ARPE-19- and HUVEC-derived VR#1814 virus preparations, following 132 passages in HUVEC (Figure 4) (in contrast to the total blocking effect of the mAb on epithelial/endothelial cells), is likely to be due to the lower initial concentration used, as suggested by other reports [10]. However, when VR#1814 ARPE-19 and HUVEC preparations were obtained starting directly from HELF/27 passage, Nrp-2 showed its complete blocking activity in both cell cultures, thus documenting that the interaction of Nrp-2 with its ligand may vary following prolonged virus propagation. In addition, the lower effect of Nrp-2 may be due to the fact that the interaction between the receptor and its ligand occurs within the endosome, so that soluble Nrp-2 cannot inhibit the infection similar to PDGFRα on HELF.

Some years ago it was shown that laboratory strains of HCMV differ in the TC/PC ratio in the virion envelope [18], and are associated with different expression levels of gO among HCMV strains [19]. However, the genetic correlates that give rise to differing PC vs. TC levels are still unclear. Recently, it has been shown that genetic differences in gO did not contribute to the levels of TC vs. PC, but seemed to affect entry, spread and neutralization by anti-gH antibodies, suggesting epistasis with other variable loci [39].

In addition, it was reported that the homologous NAb titer in PI (NAb vs. the infecting virus) could be 8-fold to more than 64-fold different from (higher than) the heterologous NAb titer (NAb titer vs. strains recovered from other patients with PI) [40]. The conclusion of that study was that, in a vaccine perspective, a high level of cross-NAb would have reached the highest level of protection. However, attempts to ameliorate the prognosis of HCMV disease, namely in transplanted patients, by administration of Ig preparations with high antibody titers have given so far equivocal results. On the other hand, in a phase-2 randomized placebo-controlled trial, the use of a combination of two anti-HCMV mAbs, one specific for pUL128 and the other for gH, showed a reduction in both HCMV infection and disease in high-risk seronegative kidney transplant recipients [41]. In addition, in hematopoietic stem cell transplant recipients, a combination of two mAbs (anti-PC and anti-gB) showed a trend towards reduction in viral load and preemptive therapy duration [42]. Furthermore, recently in a new mouse model investigating the possibility to prevent HCMV reactivation in bone marrow transplantation of mice, it was found that viral reactivation was prevented if the immune serum administered was matched to the infecting viral strain [43]. Much larger amounts of immune serum had to be administered to protect mice from unrelated mouse CMV infections.

The role of NAb in protecting against HCMV dissemination is still somewhat controversial. However, HCMV is a cell-associated virus, which only infrequently is released in blood as cell-free virus [44], even in disseminated HCMV infections of immunocompromised patients, such as AIDS patients and transplant recipients [45]. Therefore, the protective effect of NAb should be attributed predominantly to other antibody-dependent mechanisms than direct neutralizing activity, such as antibody-dependent cellular cytotoxicity (ADCC) [46], antibody-dependent cellular phagocytosis (ADCP) [47], antibody-dependent NK cell activation [48], and antibody-dependent complement deposition (ADCD) [49]. In addition, we documented that in vitro the HCMV dissemination-inhibiting activity of NAb, both mAbs and convalescent-phase sera from PI, can be demonstrated by three different assays [11,37,50]: (i) plaque formation inhibition (PFI); (ii) leukocyte transfer inhibition (LTI), i.e., inhibition of virus transfer from infected endothelial cells to leukocytes (PMNL and M/M); and iii) syncytium formation inhibition (SFI).

The results of our study seem to document, in addition to findings of the above mentioned study by Zhou et al. [18], that not only differences do exist between two HCMV strains, but that the distribution in the virion envelope of TC and PC is a dynamic process subject to continuous variations, according to the number of cell culture passages and the type of cell culture (fibroblasts, epithelial or endothelial cells) used for virus propagation. Consequently, the results of the neutralization assays are strictly dependent on the virus strain selected and the cell substrate supporting virus production. From the diagnostic standpoint, the clinical significance of the NAb determination may be precious either for a very early detection of seroconversion or a delayed diagnosis. In either case, the result may be critical for the definition of the onset of PI in pregnancy [16] as well as the development of both therapeutic antibodies and subunit vaccines.

## 4. Materials and Methods

### 4.1. Cell Cultures

Three types of cell cultures were used for virus strain propagation and cross-Nt assays: diploid HELF, a cell line of human retinal pigmented epithelial cells (ARPE-19), and multiple preparations of primary human umbilical vein endothelial cells (HUVEC). HELF were derived from a cell strain developed in our laboratory in 1980 and used at passages 20–30. HUVECs were developed by trypsin treatment of umbilical cord veins and used at passages 2–5. Finally, ARPE-19 (ATCC CRL-2302, Manassas, VA, USA) was a spontaneously arising retinal pigmented epithelial (RPE) cell line deriving from human eye.

### 4.2. Study Population and HCMV Strains

Sequential serum samples were collected from three pregnant women with HCMV PI at the following time points after PI onset: serum#1, within 60 (31–60) days (d); serum#2, after 60 d (61–90); serum#3, after 90 d (91–120); serum#4, after 120 d (121–180); serum#5, after 180 d (181–360). The three pregnant women were reported as: (i) patient p-232, who transmitted the infection to the fetus and from whom HCMV was recovered from amniotic fluid (VR#1); (ii) patient p-236, who did not transmit the infection to the fetus, and from whom HCMV was recovered from urine (VR#2); and patient p-237, who did not either transmit the infection and from whom virus was isolated from vaginal secretions (VR#3). All three women had viral DNA quantified by real-time qPCR in each of the following three clinical samples: urine, saliva and vaginal secretions [28,29,30]. In more detail, VR#1 was detected at the peak of viral load (VL, 28,000 cp/mL) at day 92, then decreasing progressively; VR#2 reached the maximal VL (75,000 cp/mL) at day 53, then decreasing; finally, VR#3 peaked (25,000 cp/mL) at day 100, then rapidly declining. In all three cases the maximal levels of viral DNA were reached in vaginal secretions.

### 4.3. Genotyping of HCMV Strains

Genotyping of gB and gH of HCMV strains was performed using two multiplex real-time PCR assays and primers and probes specific for each genotype, as reported [31]. Genotyping of gO was determined according to Mattick et al. [32].

### 4.4. Cross-Neutralization Assays

Serial dilutions of sequential serum samples from one patient were tested on the three types (HELF, ARPE-19, and HUVEC) of cell cultures for NAb titer determination against the homologous virus (homologous NAb titer), as well as against the two heterologous HCMV strains (heterologous NAb titers). Due to the different replication rate of the three virus strains in the three cell culture systems, each cell-free virus preparation was titrated in each of the three types of cell cultures, in order to use a comparable number of viral infectious units for the different cross-Nt assays. In addition, the same series of sequential serum samples were tested against our reference HCMV strain VR#1814 (passaged in the same three cell culture systems) to determine the relevant heterologous NAb titers. The 50% NAb titer was considered the serum sample dilution neutralizing 50% or more of virus inoculum (~50–100 PFU) [16].

### 4.5. Inhibition Assays

The same procedure used for the NAb titer determination was employed for the performance of inhibition assays of PC and TC in the same virus preparations used for cross-Nt assays. For inhibition assays of PC and TC, different virus preparations at a dilution containing 50–100 PFU/50 μL were incubated with serial dilutions of an equal volume of: (i) a human mAb (9I6) directed to all three UL128–131 locus (*UL128L*) products of PC (11,14,37); (ii) the recombinant soluble human PDGFR-α (R&D Systems, Minneapolis, MN, USA), the TC receptor; (iii) the recombinant soluble human PC receptor, the human neuropilin-2 (Nrp-2) Fc Chimera (R&D Systems); (iv) the TGFβRIII (R&D Systems). In both cases (neutralization and inhibitory assays), after 1 h incubation at 37 °C in a 5% CO_2_ atmosphere, 50 μL of each mixture dilution were transferred into confluent monolayers of HELF, ARPE-19 or HUVEC cells in 96-well flat bottom microplates, centrifuged at 600× *g* for 30 min and incubated for 2 h at 37 °C. Then, microplates were washed and fed with 100 μL medium/well. After 48 h cells were fixed, permeabilized and stained with a murine anti-p72 mAb [17] followed by incubation with anti-mouse IgG Alexa Fluor 594 (Thermo Fisher Scientific, Waltman, MA, USA). Finally, DAPI at a concentration of 200 ng/mL (Thermo Fisher Scientific) was added as a counterstain. Infected cells were counted with a cell-imaging microplate reader (Cytation 3, Biotek, Winovski, VT, USA, software Gen5 2.09). The 50% inhibitory concentration (IC_50_) was calculated with Prism 8.3.0 (Graph Pad Software, San Diego, CA, USA).

### 4.6. Expression of PC and TC on Virus Preparations

The relative expression of PC and TC on cell-free virus preparations obtained from infected cell culture supernatants was determined by immunoblot analysis [51]. Virus stocks containing an amount of viral DNA of 10^8^ copies/mL were used. We attempted to normalize virus preparations on the major capsid protein (MCP) content. However, ARPE-derived virus stocks had a significantly lower amount of detectable MCP; therefore, it was possible to normalize only HELF- and HUVEC-derived virus stocks. On the other hand, it was possible to normalize MCP in virus preparations obtained after a single back passage to HELF from ARPE-19 and HUVEC (Figure 5D).

### 4.7. Immunoblotting

Supernatants containing HCMV virions were cleared of cellular debris by centrifugation at 6000× *g* for 10 min, then concentrated in 300,000 MWCO Vivaspin 20 filters (Sartorius, Inc., Göttingen, Germany) to approximately 10^9^–10^10^ DNA copies/mL. Virions were pelleted by centrifugation at 20,000× *g* for 1 h followed by 2 washes with PBS. Pellets were solubilized in 2% SDS-20 mM Tris-buffered saline (TBS, pH 6.8) and insoluble material was removed by centrifugation at 16,000× *g* for 15 min. For reducing blots, dithiothreitol (DTT) was added to extracts to a final concentration of 25 mM.

Samples were boiled for 10 min prior to separation by SDS-PAGE using 4–20% precast gels (BioRad, Hercules, CA, USA). Proteins were transferred onto polyvinylidene difluoride (PVDF) membranes (Whatman) in a buffer containing 25 mM Tris, 192 mM Glycine (pH 8.3) plus 10% methanol. Transferred proteins were probed with anti-MCP mAb provided by Bill Britt (University of Alabama, Birmingham, AL, USA) or rabbit polyclonal sera against HCMV gL (kindly provided by David Johnson, Oregon Health and Sciences University, Portland, OR, USA) [27], then by anti-rabbit or anti-mouse secondary antibodies conjugated with horseradish peroxidase (Sigma-Aldrich, St. Louis, MO, USA), and Pierce ECL-Western blotting substrate (Thermo Fisher Scientific). Chemiluminescence was detected using a Bio-Rad ChemiDoc MP imaging system. Band densities were quantified using Bio-Rad Image Lab v 5.1 [51].

### 4.8. Determination of IgG Antibodies to PC, TC, and gB by ELISA

Following preparation and purification of the three gCs, as reported [11], 96-well polystyrene plates were coated with an in-house developed anti-gH mAb or an anti-gB mAb [14]. Then, ELISA plates were incubated with cell culture supernatants containing PC, TC or gB from transfected cells. Finally, human sera were added starting from the 1:100 dilution prior to adding the horseradish peroxidase-labeled goat IgG fraction to human IgG and the substrate solution. Cut-offs (OD) of 0.1 for PC and TC, and 0.3 for gB, were determined using HCMV-seronegative and -seropositive sera from healthy blood donors.

## Figures and Tables

**Table 1:** Homologous and heterologous NAb titers of p-232 vs. VR#1 (homologous) and p-236 and p-237 vs. VR#1 (heterologous).

NAb Titers	VR#1/HELF/14			VR#1/HELF/8-ARPE/3			VR#1/HELF/11-HUVEC/20		
	HELF	ARPE	HUVEC	HELF	ARPE	HUVEC	HELF	ARPE	HUVEC
Homologous titer									
p-232 (VR#1)									
Serum #1	<10	NA	NA	10	1280	80	<10	2560	640
#2	<10	NA	NA	80	1280	160	20	2560	1280
#3	10	NA	NA	160	1280	640	40	2560	5120
#4	320	NA	NA	320	1280	640	160	10,240	10,240
#5	2560	NA	NA	2560	1280	1280	2560	20,480	20,480
Heterologous titers									
p-236 (VR#2)									
Serum #1	<10	NA	NA	10	160	40	<10	640	160
#2	<10	NA	NA	40	1280	80	<10	640	640
#3	20	NA	NA	160	2560	1280	160	1280	2560
#4	80	NA	NA	320	2560	2560	160	5120	5120
#5	160	NA	NA	1280	1280	640	320	5120	1280
p-237 (VR#3)									
Serum #1	<10	NA	NA	10	160	20	10	640	160
#2	<10	NA	NA	20	640	80	40	640	320
#3	10	NA	NA	40	1280	640	80	1280	640
#4	20	NA	NA	160	1280	2560	160	1280	160
#5	80	NA	NA	160	640	640	320	1280	160

Serum #1, <60 (31–60) days (d) post infection (p.i.) onset; serum #2, >60 (61–90) d p.i.; serum #3, >90 (91–120) d p.i.; serum #4, >120 (121–180) d p.i.; serum #5, >180 (181–360) d p.i.; NA, not applicable.

**Table 2:** Homologous and heterologous NAb titers of p-236 vs. VR#2 (homologous) and p-232 and p-237 vs. VR#2 (heterologous).

NAb Titers	VR#2/HELF/12			VR#2/HELF/9-ARPE/4			VR#2/HELF/4-HUVEC/14		
	HELF	ARPE	HUVEC	HELF	ARPE	HUVEC	HELF	ARPE	HUVEC
Homologous titer									
p-236 (VR#2)									
Serum #1	<10	NA	NA	<10	1280	320	80	320	640
#2	<10	NA	NA	80	2560	640	80	640	640
#3	160	NA	NA	1280	10,240	5120	320	10,240	2560
#4	640	NA	NA	5120	10,240	5120	320	20,480	5120
#5	2560	NA	NA	20,480	10,240	10,240	640	10,240	10,240
Heterologous titers									
p-232 (VR#1)									
Serum #1	<10	NA	NA	40	1280	160	40	640	160
#2	<10	NA	NA	160	1280	640	40	640	640
#3	10	NA	NA	1280	1280	640	80	640	640
#4	40	NA	NA	2560	2560	2560	160	2560	2560
#5	160	NA	NA	5120	5120	2560	320	2560	2560
p-237 (VR#3)									
Serum #1	<10	NA	NA	<10	40	40	40	160	160
#2	<10	NA	NA	160	640	320	160	160	320
#3	10	NA	NA	1280	2560	1280	320	640	640
#4	160	NA	NA	1280	2560	2560	160	640	640
#5	160	NA	NA	1280	2560	1280	160	640	640

Serum #1, <60 (31–60) days (d) post infection (p.i.) onset; serum #2, >60 (61–90) d p.i.; serum #3, >90 (91–120) d p.i.; serum #4, >120 (121–180) d p.i.; serum #5, >180 (181–360) d p.i.; NA, not applicable.

**Table 3:** Homologous and heterologous NAb titers of p-237 vs. VR#3 (homologous) and p-232 and p-236 vs. VR#3 (heterologous).

NAb Titers	VR#3/HELF/12			VR#3/HELF/12-ARPE/4			VR#3/HELF/10-HUVEC/8		
	HELF	ARPE	HUVEC	HELF	ARPE	HUVEC	HELF	ARPE	HUVEC
Homologous titer									
p-237 (VR#3)									
Serum #1	<10	NA	NA	<10	320	40	<10	160	40
#2	10	NA	NA	<10	320	80	10	320	80
#3	20	NA	NA	80	640	160	40	640	160
#4	80	NA	NA	160	640	160	320	1280	160
#5	1280	NA	NA	1280	640	160	2560	640	160
Heterologous titers									
p-232 (VR#1)									
Serum #1	<10	NA	NA	10	1280	160	<10	1280	160
#2	20	NA	NA	10	1280	640	10	640	160
#3	10	NA	NA	10	1280	640	10	320	160
#4	10	NA	NA	80	1280	320	40	640	160
#5	160	NA	NA	1280	1280	320	320	2560	320
p-236 (VR#2)									
Serum #1	<10	NA	NA	<10	1280	40	<10	2560	320
#2	20	NA	NA	<10	1280	160	<10	5120	320
#3	40	NA	NA	40	1280	1280	80	2560	640
#4	40	NA	NA	80	640	1280	80	5120	640
#5	160	NA	NA	160	640	320	160	640	640

Serum #1, <60 (31–60) days (d) post infection (p.i.) onset; serum #2, >60 (61–90) d p.i.; serum #3, >90 (91–120) d p.i.; serum #4, >120 (121–180) d p.i.; serum #5, >180 (181–360) d p.i.; NA, not applicable.

**Table 4:** Heterologous NAb titers of p-232, p-236 and p-237 vs. VR#1814.

NAb Titers	VR#1814/HELF/27			VR#1814/HUVEC/132-ARPE/8			VR#1814/HUVEC/132		
	HELF	ARPE	HUVEC	HELF	ARPE	HUVEC	HELF	ARPE	HUVEC
Heterologous titers									
p-232 (VR#1)									
Serum #1	<10	NA	NA	<10	1280	320	<10	2560	320
#2	<10	NA	NA	<10	1280	320	<10	2560	640
#3	<10	NA	NA	<10	640	160	10	1280	320
#4	40	NA	NA	20	640	160	20	1280	320
#5	640	NA	NA	160	1280	320	320	2560	320
p-236 (VR#2)									
Serum #1	10	NA	NA	<10	2560	320	<10	5120	640
#2	10	NA	NA	<10	1280	640	<10	2560	1280
#3	40	NA	NA	20	1280	640	40	2560	1280
#4	40	NA	NA	20	1280	640	80	5120	1280
#5	80	NA	NA	80	1280	640	80	5120	1280
p-237 (VR#3)									
Serum #1	<10	NA	NA	<10	320	40	<10	1280	160
#2	10	NA	NA	<10	320	160	<10	1280	160
#3	20	NA	NA	<10	640	320	10	2560	320
#4	80	NA	NA	<10	640	160	20	1280	640
#5	320	NA	NA	40	640	160	80	1280	160

Serum #1, <60 (31–60) days (d) post infection (p.i.) onset; serum #2, >60 (61–90) d p.i.; serum #3, >90 (91–120) d p.i.; serum #4, >120 (121–180) d p.i.; serum #5, >180 (181–360) d p.i.; NA, not applicable.

## Data Availability

Raw data are available from the authors on reasonable request.

## Supplementary Materials

The following supporting information can be downloaded at: https://www.mdpi.com/article/10.3390/ijms24054417/s1.

## References

[1] Chou S.W., Dennison K.M. (1991). Analysis of interstrain variation in cytomegalovirus glycoprotein B sequences encoding neutralization-related epitopes. J. Infect. Dis..

[2] Chou S.W. (1992). Molecular epidemiology of envelope glycoprotein H of human cytomegalovirus. J. Infect. Dis..

[3] Urban M., Britt W., Mach M. (1992). The dominant linear neutralizing antibody-binding site of glycoprotein gp86 of human cytomegalovirus is strain-specific. J. Virol..

[4] Burkhardt C., Himmelein S., Britt W., Winkler T., Mach M. (1992). Glycoprotein N subtypes of human cytomegalovirus induce a strain-specific antibody response during natural infection. J. Gen. Virol..

[5] Pati S.K., Novak Z., Purser M., Arora N., Mach M., Britt W.J., Boppana S.B. (2012). Strain-specific neutralizing antibody responses against human cytomegalovirus envelope glycoprotein N. Clin. Vacc. Immunol..

[6] Wille P.T., Knoche A.J., Nelson J.A., Jarvis M.A., Johnson D.C. (2010). A human cytomegalovirus gO-null mutant fails to incorporate gH/gL into the virion envelope and is unable to enter fibroblasts and epithelial and endothelial cells. J. Virol..

[7] Hahn G., Revello M.G., Patrone M., Percivalle E., Campanini G., Sarasini A., Wagner M., Gallina A., Milanesi G., Koszinowski U. (2004). Human cytomegalovirus UL131-128 genes are indispensable for virus growth in endothelial cells and virus transfer to leukocytes. J. Virol..

[8] Ryckman B.J., Chase M.C., Johnson D.C. (2008). HCMV gH/gL/UL128-131 interferes with virus entry into epithelial cell: Evidence for type-specific receptors. Proc. Natl. Acad. Sci. USA.

[9] Kabanova A., Marcandalli J., Zhou T., Bianchi S., Baxa U., Tsybovski Y., Lilleri D., Silacci-Fregni C., Foglierini M., Fernandez-Rodriguez B.M. (2016). Platelet-derived growth factor-alpha receptor is the cellular receptor for human cytomegalovirus gHgLgO trimer. Nat. Microbiol..

[10] Martinez-Martin N., Marcandalli J., Huang C.S., Arthur C.P., Perotti M., Foglierini M., Ho H., Dosey A.M., Shriver S., Payandeh J. (2018). An unbiased screen for human cytomegalovirus identifies neuropilin-2 as a central viral receptor. Cell.

[11] Lilleri D., Kabanova A., Revello M.G., Percivalle E., Sarasini A., Genini E., Sallusto F., Lanzavecchia A., Corti D., Gerna G. (2013). Fetal human cytomegalovirus transmission correlates with delayed maternal antibodies to gH/gL/pUL128-130-131 complex during primary infection. PLoS ONE.

[12] Kabanova A., Perez L., Lilleri D., Marcandalli J., Agatic G., Becattini S., Preite S., Fuschillo D., Percivalle E., Sallusto F. (2014). Antibody-driven design of a human cytomegalovirus gHgLpUL128L subunit vaccine that selectively elicits potent neutralizing antibodies. Proc. Natl. Acad. Sci. USA.

[13] Fouts A.E., Chan P., Stefan J.P., Vandler R., Feierbach B. (2012). Antibodies against the gH/gL/UL128/UL130/UL131 complex comprise the majority of the anti-cytomegalovirus (anti-CMV) neutralizing antibody response in CMV hyperimmune globulin. J. Virol..

[14] Macagno A., Bernasconi N., Vanzetta F., Dander E., Sarasini A., Revello M.G., Gerna G., Sallusto F., Lanzavecchia A. (2010). Isolation of human monoclonal antibodies that potently neutralize HCMV infection by targeting different epitopes on the gH/gL/UL128-131A complex. J. Virol..

[15] Vanarsdall A.L., Chin A.L., Liu J., Jardetzky T.S., Mudd J.O., Orloff S.L., Streblow D., Mussi-Pinhata M.M., Yamamoto A.Y., Duarte G. (2019). HCMV trimer-and pentamer-specific antibodies synergize for virus neutralization but do not correlate with congenital transmission. Proc. Natl. Acad. Sci. USA.

[16] Lilleri D., Gerna G., Furione M., Zavattoni M., Spinillo A. (2016). Neutralizing and ELISA IgG antibodies to human cytomegalovirus glycoprotein complexes may help date the onset of primary infection in pregnancy. J. Clin. Virol..

[17] Gerna G., Sarasini A., Patrone M., Percivalle E., Fiorina L., Campanini G., Gallina A., Baldanti F., Revello M.G. (2008). Human cytomegalovirus serum neutralizing antibodies block virus infection of endothelial/epithelial cells, but not fibroblasts, early during primary infection. J. Gen. Virol..

[18] Zhou M., Yu Q., Wechsler A., Ryckman B.J. (2013). Comparative analysis of gO isoforms reveals that strains of human cytomegalovirus differ in the ratio of gH/gL/gO and gH/gL/UL128-131 in the virion envelope. J. Virol..

[19] Zhang L., Zhou M., Stanton R., Kamil J., Ryckman B.J. (2018). Expression levels of glycoprotein O (gO) vary between strains of human cytomegalovirus, influencing the assembly of gH/gL complexes and virion infectivity. J. Virol..

[20] Zhou M., Lanchy J.-M., Ryckman B.J. (2015). Human cytomegalovirus gH/gL/gO promotes the fusion step of entry into all cell types, whereas gH/gL/UL128-131 broadens virus tropism through a distinct mechanism. J. Virol..

[21] Gerna G., Percivalle E., Lilleri D., Lozza L., Fornara C., Hahn G., Baldanti F., Revello M.G. (2005). Dendritic-cell infection by human cytomegalovirus is restricted to strains carrying functional UL131-128 genes and mediates efficient viral antigen presentation to CD8^+^ T cells. J. Gen. Virol..

[22] Liu J., Jardetzky T.S., Chin A.L., Johnson D.C., Vanarsdall A.L. (2018). The human cytomegalovirus trimer and pentamer promote sequential steps in entry into epithelial and endothelial cells at cell surfaces and endosomes. J. Virol..

[23] Nguyen C.C., Kamil J.P. (2018). Pathogen at the gates: Human cytomegalovirus entry and cell tropism. Viruses.

[24] Kschonsak M., Rougè L., Arthur C.P., Hoangdung H., Patel N., Kim I., Johnson M.C., Kraft E., Rohou A.L., Gill A. (2021). Structures of HCMV trimer reveal the basis for receptor recognition and cell entry. Cell.

[25] Borza C.M., Hutt-Fletcher L.M. (2002). Alternate replication in B cells and epithelial cells switches tropism of Epstein-Barr virus. Nat. Med..

[26] Scrivano L., Sinzger C., Nitschko H., Koszinowski U.H., Adler B. (2011). HCMV spread and cell tropism are determined by distinct virus populations. PLoS Pathog..

[27] Schultz E.P., Lanchy J.-M., Day L.Z., Yu Q., Peterson C., Preece J., Ryckman B.J. (2020). Specialization for cell-free or cell-to-cell spread of Bac-cloned human cytomegalovirus strains is determined by factors beyond the UL128-131 and RL13 loci. J. Virol..

[28] Furione M., Rognoni V., Cabano E., Baldanti F. (2012). Kinetics of human cytomegalovirus (HCMV) DNAemia in transplanted patients expressed in international units as determined with the Abbott RealTime CMV assay and an in-house assay. J. Clin. Virol..

[29] Revello M.G., Furione M., Rognoni V., Arossa A., Gerna G. (2014). Cytomegalovirus DNAemia in pregnant women. J. Clin. Virol..

[30] Suarez N.M., Wilkie G.S., Hage E., Camiolo S., Holton M., Hughes J., Maabar M., Vattipally S.B., Dhingra A., Gompels U.A. (2019). Human cytomegalovirus genomes sequenced directly from clinical material: Variation, multiple-strain infection, recombination, and gene loss. J. Infect. Dis..

[31] De Vries J.J., Wessel E., Korver A.M., van der Eijk A.A., Rusman L.G., Kroes A.C., Vossen A.C. (2012). Rapid genotyping of cytomegalovirus in dried blood spots by multiplex real-time PCR assays targeting the envelope glycoprotein B and gH genes. J. Clin. Microbiol..

[32] Mattick C., Dewin D., Polley S., Sevilla-Reyes E., Pignatelli S., Rawlinson W., Wilkindson G., Dal Monte P., Gompels U.A. (2004). Linkage of human cytomegalovirus glycoprotein gO variant groups identified from worldwide clinical isolates with gN genotypes, implications for disease associations and evidence for N-terminal sites of positive selection. Virology.

[33] Revello M.G., Baldanti F., Percivalle E., Sarasini A., De-Giuli L., Genini E., Lilleri D., Labò N., Gerna G. (2001). In vitro selection of human cytomegalovirus variants unable to transfer virus and virus products from infected cells to polymorphonuclear leukocytes and to grow in endothelial cells. J. Gen. Virol..

[34] Murrell I., Bedford C., Ladell K., Miners K.L., Price D.A., Tomasec P., Wilkinson G.W.G., Stanton R.J. (2017). The pentameric complex drives immunologically covert cell-cell transmission of wild-type human cytomegalovirus. Proc. Natl. Acad. Sci. USA.

[35] Al Qaffas A., Camiolo S., Vo M., Aguiar A., Ourahmane A., Sorono M., Davison A.J., McVoy M.A., Hertel L. (2021). Genome sequences of human cytomegalovirus strain TB40/E variants propagated in fibroblasts and epithelial cells. Virol. J..

[36] Vo M., Aguiar A., McVoy M.A., Hertel L. (2020). Cytomegalovirus Strain TB40/E Restrictions and Adaptations to Growth in ARPE-19 Epithelial Cells. Microorganisms.

[37] Lilleri D., Kabanova A., Lanzavecchia A., Gerna G. (2012). Antibodies against neutralization epitopes of human cytomegalovirus gH/gL/pUL128-130-131 complex and virus spreading may correlate with virus control in vivo. J. Clin. Immunol..

[38] E X., Meraner P., Lu P., Perreira J.M., Aker A.M., McDougall W.M., Zhu R., Chan G.C., Gerstein R.M., Caposio P. (2019). OR14I1 is a receptor for the human cytomegalovirus pentameric complex and defines viral epithelial cell tropism. Proc. Natl. Acad. Sci. USA.

[39] Day L.Z., Stegman C., Schultz E.P., Lanchy J.-M., Yu Q., Ryckman B.J. (2020). Polymorphisms in human cytomegalovirus glycoprotein O (gO) exert epistatic influences on cell-free and cell-to-cell spread and antibody neutralization on gH epitopes. J. Virol..

[40] Klein M., Schoppel K., Amvrossiadis N., Mach M. (1999). Strain-specific neutralization of human cytomegalovirus isolates by human sera. J. Virol..

[41] Ishida J.H., Patel A., Mehta A.K., Gatault P., McBride J.M., Burgess T., Derby M.A., Snydman D.R., Emu B., Feierbach B. (2017). Phase 2 Randomized, Double-Blind, Placebo-Controlled Trial of RG7667, a Combination Monoclonal Antibody, for Prevention of Cytomegalovirus Infection in High-Risk Kidney Transplant Recipients. Antimicrob. Agents Chemother..

[42] Maertens J., Logan A.C., Jang J., Long G., Tang J.L., Hwang W.Y.K., Koh L.P., Chemaly R., Gerbitz A., Winkler J. (2020). Phase 2 Study of Anti-Human Cytomegalovirus Monoclonal Antibodies for Prophylaxis in Hematopoietic Cell Transplantation. Antimicrob. Agents Chemother..

[43] Martins J.P., Andoniou C.E., Fleming P., Kuns R.D., Schuster I.S., Voigt V., Daly S., Varelias A., Tey S.-K., Degli-Esposti M.A. (2019). Strain-specific antibody therapy prevents cytomegalovirus reactivation after transplantation. Science.

[44] Gilbert G.L., Hayes K., Hudson I.L., James J. (1989). Prevention of transfusion-acquired cytomegalovirus infection in infants by blood filtration to remove leucocytes. Neonatal Cytomegalovirus Infection Study Group. Lancet.

[45] Gerna G., Zipeto D., Percivalle E., Parea M., Revello M.G., Maccario R., Peri G., Milanesi G. (1992). Human cytomegalovirus infection of the major leukocyte subpopulations and evidence for initial viral replication in polymorphonuclear leukocytes from viremic patients. J. Infect. Dis..

[46] Gomez-Roman V.R., Florese R.H., Patterson L.J., Peng B., Venzon D., Aldrich K., Robert-Guroff M. (2006). A simplified method for rapid fluorometric assessment of antibody-dependent cell-mediated cytotoxicity. J. Immunol. Methods.

[47] Ackerman M.E., Moldt B., Wyatt R.T., Dugast A.-S., McAndrew E., Tsoukas S., Jost S., Berger C., Sciarangella G., Liu Q. (2011). A robust, high-throughput assay to determine the phagocyte activity of clinical antibody samples. J. Immunol. Methods.

[48] Chung A.W., Ghebremichael M., Robinson H., Brown E., Choi I., Lane S., Dugast A.-S., Schoen M.K., Rolland M., Suscovich T.J. (2014). Polyfunctional Fc-effector profiles mediated by IgG subclass selection distinguish RV144 and VAX003 vaccines. Sci. Transl. Med..

[49] Chung A.W., Kumar M.P., Arnold K.B., Yu W.H., Schoen M.K., Dunphy L.J., Suscovich T.J., Frahm N., Linde C., Mahan A.E. (2015). Dissecting polyclonal vaccine-induced humoral immunity against HIV using systems serology. Cell.

[50] Gerna G., Percivalle E., Perez L., Lanzavecchia A., Lilleri D. (2016). Monoclonal antibodies to different components of the human cytomegalovirus (HCMV) pentamer gH/gL/pUL128L and trimer gH/gL/gO as well as antibodies elicited during primary HCMV infection prevent epithelial cell syncytium formation. J. Virol..

[51] Schultz E.P., Yu Q., Stegmann C., Day L.Z., Lanchy J.M., Ryckman B.J. (2021). Mutagenesis of Human Cytomegalovirus Glycoprotein L Disproportionately Disrupts gH/gL/gO over gH/gL/pUL128-131. J. Virol..

