# Supplementary material for: Fibroblast, Epithelial and Endothelial Cell-Derived Human Cytomegalovirus Strains Display Distinct Neutralizing Antibody Responses and Varying Levels of gH/gL Complexes
Source: Int J Mol Sci. 2023 Feb 23;24(5):4417. doi: 10.3390/ijms24054417 (PMC10003051; doi:10.3390/ijms24054417)
Supplement: Supplementary file 1 [file ijms-24-04417-s001.zip › ijms-2149872-supplementary.pdf]

Table S1. 50% inhibitory concentrations (ICs_{50}) of mAb 9I6 neutralizing PC, sNRp-2, sPDGFR-α and sTGFβRIII in VR#1, VR#2 and VR#3 virus preparations obtained in HELF, ARPE-19 and HUVEC cell substrates.

virus preparation	inhibitor	IC_{50} (μg/ml)		
		**HELF**	**ARPE-19**	**HUVEC**
**VR#1 HELF/14**	**mAb 9I6**	>1	NA	NA
	**sNrp-2**	>5	NA	NA
	**sPDGFR-α**	$1.03x10^{-2}$	NA	NA
	**sTGFβRIII**	4.44	NA	NA
**VR#1 HELF/8-ARPE/3**	**mAb 9I6**	>1	$6.53x10^{-5}$	$1.89x10^{-3}$
	**sNrp-2**	$1.21x10^{-1}$	$9.40x10^{-4}$	$2.06x10^{-4}$
	**sPDGFR-α**	$3.84x10^{-3}$	$8.67x10^{-3}$	>1
	**sTGFβRIII**	9.12	$5.61x10^{-3}$	>10
**VR#1 HELF/8-ARPE/6 HELF/1**	**mAb 9I6**	>1	NA	NA
	**sNrp-2**	$8.85x10^{-2}$	NA	NA
	**sPDGFR-α**	$5.62x10^{-3}$	NA	NA
	**sTGFβRIII**	>10	NA	NA
**VR#1 HELF/11-HUVEC/20**	**mAb 9I6**	$7.40x10^{-3}$	$4.33x10^{-4}$	$4.18x10^{-4}$
	**sNrp-2**	$8.59x10^{-2}$	$4.68x10^{-3}$	$4.09x10^{-2}$
	**sPDGFR-α**	$1.57x10^{-3}$	$2.07x10^{-3}$	>1
	**sTGFβRIII**	6.79	0.39	>10
**VR#1 HELF/11-HUVEC/20 HELF/1**	**mAb 9I6**	$1.88x10^{-2}$	NA	NA
	**sNrp-2**	$4.89x10^{-2}$	NA	NA
	**sPDGFR-α**	$5.95x10^{-3}$	NA	NA
	**sTGFβRIII**	$7.7x10^{-1}$	NA	NA
**VR#2 HELF/12**	**mAb 9I6**	>1	NA	NA
	**sNrp-2**	>5	NA	NA
	**sPDGFR-α**	$4.89x10^{-3}$	NA	NA
	**sTGFβRIII**	2.70	NA	NA
**VR#2 HELF/9-ARPE/4**	**mAb 9I6**	$6.99x10^{-4}$	$2.76x10^{-4}$	$7.50x10^{-4}$
	**sNrp-2**	$2.84x10^{-1}$	$9.26x10^{-3}$	$1.18x10^{-1}$
	**sPDGFR-α**	$1.04x10^{-2}$	$7.06x10^{-3}$	>1
	**sTGFβRIII**	$1.09x10^{-2}$	3.33	1.07
**VR#2 HELF/9-ARPE/6 HELF/1**	**mAb 9I6**	>1	NA	NA
	**sNrp-2**	$3.88x10^{-1}$	NA	NA
	**sPDGFR-α**	$7.55x10^{-3}$	NA	NA
	**sTGFβRIII**	1.48	NA	NA
**VR#2 HELF/4-HUVEC/14**	**mAb 9I6**	$5.97x10^{-4}$	$3.16x10^{-4}$	$3.56x10^{-4}$
	**sNrp-2**	$4.07x10^{-2}$	$4.07x10^{-1}$	$1.50x10^{-1}$
	**sPDGFR-α**	$2.31x10^{-2}$	>1	>1
	**sTGFβRIII**	>10	>10	>10
**VR#2 HELF/4-HUVEC/14 HELF/1**	**mAb 9I6**	>1	NA	NA
	**sNrp-2**	5.36	NA	NA
	**sPDGFR-α**	$2.27x10^{-3}$	NA	NA
	**sTGFβRIII**	3.40	NA	NA
**VR#3 HELF/12**	**mAb 9I6**	>1	NA	NA
	**sNrp-2**	>5	NA	NA
	**sPDGFR-α**	$4.61x10^{-3}$	NA	NA
	**sTGFβRIII**	9.90	NA	NA
**VR#3 HELF/12-ARPE/4**	**mAb 9I6**	>1	$9.82x10^{-5}$	$3.40 x10^{-4}$
	**sNrp-2**	$3.22 x10^{-2}$	$1.14x10^{-2}$	$5.79x10^{-2}$
	**sPDGFR-α**	$2.31x10^{-2}$	$1.79x10^{-2}$	>1
	**sTGFβRIII**	$1.04x10^{-2}$	5.94	4.94
**VR#3 HELF/12-ARPE/4-HELF/1**	**mAb 9I6**	$5.6x10^{-2}$	NA	NA
	**sNrp-2**	$2.79 x10^{-1}$	NA	NA
	**sPDGFR-α**	$2.1x10^{-2}$	NA	NA
	**sTGFβRIII**	$2.40x10^{-1}$	NA	NA

virus preparation	inhibitor	IC50 (μg/ml)		
		**HELF**	**ARPE-19**	**HUVEC**
**VR#3 HELF/10-HUVEC/8**	**mAb 9I6**	>1	$1.51x10^{-4}$	$1.01x10^{-4}$
	**sNrp-2**	$2.73x10^{-2}$	$7.50x10^{-3}$	$3.92x10^{-1}$
	**sPDGFR-α**	$9.63x10^{-3}$	$1.41x10^{-2}$	>1
	**sTGFβRIII**	>10	$1.92x10^{-2}$	>10
**VR#3 HELF/10-HUVEC/8 HELF/1**	**mAb 9I6**	>1	NA	NA
	**sNrp-2**	2.35	NA	NA
	**sPDGFR-α**	$5x10^{-2}$	NA	NA
	**sTGFβRIII**	7.39	NA	NA
**VR#1814 HELF/27**	**mAb 9I6**	>1	NA	NA
	**sNrp-2**	>5	NA	NA
	**sPDGFR-α**	$3.32x10^{-2}$	NA	NA
	**sTGFβRIII**	>10	NA	NA
**VR#1814 HELF/27-ARPE/5**	**mAb 9I6**	$1.01x10^{-3}$	$4.58x10^{-5}$	$1.96 x10^{-3}$
	**sNrp-2**	$1.26x10^{-2}$	$8.30x10^{-3}$	$5.36x10^{-2}$
	**sPDGFR-α**	$1.01x10^{-2}$	$1.03 x10^{-4}$	>1
	**sTGFβRIII**	$2.31x10^{-4}$	>10	>10
**VR#1814 HELF/27-HUVEC/6**	**mAb 9I6**	$2.88x10^{-2}$	$5.30x10^{-4}$	$1.76x10^{-3}$
	**sNrp-2**	$9.31x10^{-2}$	$1.44x10^{-2}$	$1.24x10^{-1}$
	**sPDGFR-α**	$2.82x10^{-2}$	$9.30x10^{-2}$	>1
	**sTGFβRIII**	>10	>10	>10
**VR#1814 HELF/60-HUVEC/132**	**mAb 9I6**	>1	$4.13x10^{-4}$	$1.55x10^{-2}$
	**sNrp-2**	>5	>5	>5
	**sPDGFR-α**	$2.88x10^{-2}$	$2.58x10^{-2}$	>1
	**sTGFβRIII**	>10	>10	>10
**VR#1814 HELF/60-HUVEC/132-ARPE/8**	**mAb 9I6**	>1	$1.32x10^{-3}$	$7.19x10^{-3}$
	**sNrp-2**	>5	>5	>5
	**sPDGFR-α**	$4.53x10^{-2}$	$1.83x10^{-2}$	>1
	**sTGFβRIII**	>10	>10	>10

NA, not applicable

Table S2. Comparison among NAb titers, on HELF, of p-236 vs VR#2 (homologous) and p-232 and p-237 *vs* VR#2 (heterologous) after a single passage of VR#2 from ARPE-19 and HUVEC to HELF.

**NAb titers**	**VR#2/ HELF/12**	**VR#2 HELF/9-**		**VR#2 HELF/4-**	
		**ARPE/4**	**ARPE/6-HELF/1**	**HUVEC/14**	**HUVEC/14-HELF/1**
**Homologous titer**					
p-236 (VR#2)					
Serum #1	<10	<10	<10	80	<10
#2	<10	80	<10	80	<10
#3	160	1,280	1,280	320	40
#4	640	5,120	1,280	320	640
#5	2,560	20,480	5,120	640	2,560
**Heterologous titers**					
p-232 (VR#1)					
Serum #1	<10	40	<10	40	<10
#2	<10	160	40	40	<10
#3	10	1,280	160	80	<10
#4	40	2,560	80	160	40
#5	160	5,120	640	320	320
p-237 (VR#3)					
Serum #1	<10	<10	<10	40	<10
#2	<10	160	<10	160	<10
#3	10	1,280	40	320	<10
#4	160	1,280	40	160	80
#5	160	1,280	160	160	80

Serum #1, <60 (31-60) days (d) post infection (p.i.) onset; serum #2, >60 (61-90) d p.i.; serum #3, >90 (91-120) d p.i.; serum #4, >120 (121-180) d p.i.; serum #5, >180 (181-360) d p.i.
